# WHA-Net: A Low-Complexity Hybrid Model for Accurate Pseudopapilledema Classification in Fundus Images

**DOI:** 10.3390/bioengineering12050550

**Published:** 2025-05-21

**Authors:** Junpeng Pei, Yousong Wang, Mingliang Ge, Jun Li, Yixing Li, Wei Wang, Xiaohong Zhou

**Affiliations:** 1 School of Health Science and Engineering, University of Shanghai for Science and Technology, Shanghai 200093, China; johnnypei168@163.com (J.P.);; 2 PLA Naval Medical Center, Naval Medical University, Shanghai 200433, China; 3 Ophthalmology, Children’s Hospital of Fudan University, National Children’s Medical Center, Shanghai 201102, China

**Keywords:** pseudopapilledema, hybrid model, auxiliary diagnosis, fundus images

## Abstract

The fundus manifestations of pseudopapilledema closely resemble those of optic disc edema, making their differentiation particularly challenging in certain clinical situations. However, rapid and accurate diagnosis is crucial for alleviating patient anxiety and guiding treatment strategies. This study proposes an efficient low-complexity hybrid model, WHA-Net, which innovatively integrates three core modules to achieve precise auxiliary diagnosis of pseudopapilledema. First, the wavelet convolution (WTC) block is introduced to enhance the model’s characterization capability for vessel and optic disc edge details in fundus images through 2D wavelet transform and deep convolution. Additionally, the hybrid attention inverted residual (HAIR) block is incorporated to extract critical features such as vascular morphology, hemorrhages, and exudates. Finally, the Agent-MViT module effectively captures the continuity features of optic disc contours and retinal vessels in fundus images while reducing the computational complexity of traditional Transformers. The model was trained and evaluated on a dataset of 1793 rigorously curated fundus images, comprising 895 normal optic discs, 485 optic disc edema (ODE), and 413 pseudopapilledema (PPE) cases. On the test set, the model achieved outstanding performance, with 97.79% accuracy, 95.55% precision, 95.69% recall, and 98.53% specificity. Comparative experiments confirm the superiority of WHA-Net in classification tasks, while ablation studies validate the effectiveness and rationality of each module’s combined design. This research provides a clinically valuable solution for the automated differential diagnosis of pseudopapilledema, with both computational efficiency and diagnostic reliability.

## 1. Introduction

**Figure 1:**
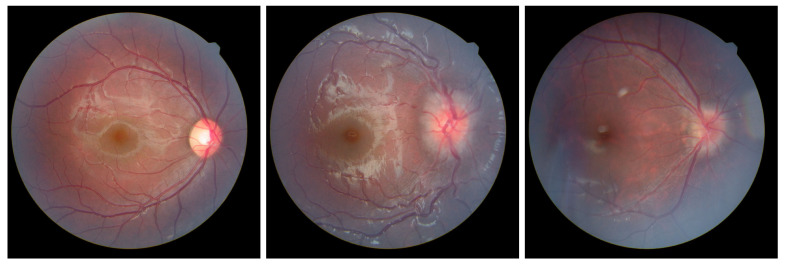
From left to right are normal optic disc, ODE, and PPE.

Optic disc edema (ODE), a common clinical sign in neuro-ophthalmology, refers to pathological swelling and elevation of the optic nerve head’s nerve fiber layer. Its etiologies include optic neuritis, ischemic/toxic/infiltrative optic neuropathies, malignant hypertension, diabetes, and papilledema (specifically denoting ODE caused by increased intracranial pressure) [1]. In this study, ODE encompasses true optic disc swelling induced by the aforementioned conditions. Pseudopapilledema (PPE) exhibits fundoscopic features resembling ODE, such as optic disc elevation and blurred margins, but demonstrates lower elevation amplitude compared to true ODE, with the absence of nerve fiber layer edema, hemorrhage, or exudates, as shown in Figure 1. PPE is unrelated to life-threatening conditions and typically arises from congenital anomalies, tilted discs, hypoplastic discs, hyperopic crowded discs, optic disc hamartomas, myelinated nerve fibers, or optic disc drusen [2]. There are substantial differences in evaluation, treatment, and prognosis between true optic disc edema and pseudopapilledema. True ODE may lead to vision loss, optic nerve damage, or death, serving as critical warning signals for life-threatening conditions such as intracranial tumors and idiopathic intracranial hypertension. It requires imaging and laboratory investigations for etiological diagnosis followed by targeted therapy. Conversely, PPE misdiagnosed as ODE results in unnecessary invasive procedures (e.g., lumbar puncture, cranial imaging) and wasteful healthcare resource utilization. Therefore, accurate and rapid differentiation between true ODE and PPE remains crucial.

The clinical diagnostic techniques for optic disc edema primarily include fundus photography, B-scan ultrasonography, fluorescein angiography (FA), fundus autofluorescence (FAF), optical coherence tomography (OCT), and OCT angiography (OCTA), supplemented by neuroimaging examinations (computed tomography (CT) and magnetic resonance imaging (MRI)) and lumbar puncture when necessary for comprehensive evaluation [3,4,5]. As a fundamental ophthalmic examination, fundus photography demonstrates notable technical advantages: compared to OCT, CT, and MRI equipment, fundus cameras exhibit lower acquisition costs, higher operational convenience, and shorter examination durations, making them widely available in ophthalmic departments across all levels of medical institutions. Achieving the rapid differentiation of pseudopapilledema through color fundus images would hold significant clinical value [6,7], as this approach could not only enhance diagnostic efficiency but also effectively prevent patients from undergoing invasive procedures and incurring high medical costs. This technology holds particular clinical significance in pediatric ophthalmology, where pseudopapilledema is more prevalent [8], as neuroimaging examinations in pediatric patients often require sedation, significantly elevating procedural complexity and associated risks.

Currently, deep-learning-based artificial intelligence (AI) technology is widely applied in medical image diagnostics. Models such as convolutional neural networks (CNNs), recurrent neural networks (RNNs), graph neural networks (GNNs), and Transformers are employed to process medical images by extracting color, luminance, shape, and structural features [9,10], enabling the precise segmentation of organs/lesions or disease classification through subtle feature differences. In ophthalmology, deep learning has been utilized for the automated detection of diabetic retinopathy and glaucoma from fundus images [11,12]. Although research on deep learning for pseudopapilledema diagnosis remains limited, these techniques have demonstrated promising potential in optic disc edema detection and grading.

This study proposes the development of a deep-learning system based on color fundus photographs for rapid and accurate differentiation of pseudopapilledema. The system can be deployed on hospital desktop fundus cameras or portable handheld devices to enable early screening, assisting ophthalmologists in making timely clinical decisions while providing medical referral recommendations for physicians without ophthalmic expertise and general users, thereby reducing diagnostic costs and optimizing healthcare resource allocation. For practical application scenarios, we designed a novel lightweight diagnostic system with low computational complexity that adapts to resource-constrained devices. Experimental results demonstrate that the proposed model achieves effective classification of pseudopapilledema with lower parameter counts and computational complexity, showing significant clinical value.The main contributions of this work include the following:

1. Construction of a multi-source dataset comprising normal optic discs, true optic disc edema, and pseudopapilledema images.
2. Proposal of the WHA-Net lightweight architecture, which delivers competitive classification accuracy without pretraining, despite limited pseudopapilledema data availability.
3. To enhance vessel and optic disc detail feature extraction while mitigating image noise interference, we introduced a wavelet convolution module that performs multi-level wavelet transforms and convolution on images, capturing richer image information compared to standard convolutional operations. Additionally, the model integrates a HAIR module to strengthen the perception of critical features such as vascular morphology, hemorrhages, and exudates, while maintaining low computational complexity.
4. To address the high computational complexity of self-attention mechanisms, we propose the Agent-MViT block, which replaces the Softmax attention in the original transformer module with agent attention. This achieves linear-complexity representation of global features, including vascular distribution patterns, optic disc color, and edema extent, while reducing computational overhead.

## 2. Related Works

In this section, we will briefly review literature and methods related to pseudopapilledema classification tasks, including the following aspects: optic disc disease classification tasks, CNN–Transformer hybrid models, attention mechanisms, and wavelet transforms.

### 2.1. Fundus Diseases Classification

The advancement of deep-learning technology has positioned retinal image-based automated detection and diagnosis of fundus diseases as a prominent research focus [13]. Researchers have explored disease diagnosis using both single-modality and multimodal retinal imaging datasets [14]. Notably, several advanced deep-learning algorithms have recently been developed for critical clinical applications, including differential diagnosis between pseudopapilledema and normal optic discs/other optic disc pathologies, discrimination of non-arteritic anterior ischemic optic neuropathy (NAION) from pseudopapilledema, and the precise grading of papilledema [15].

In 2019, Jin et al. [16] developed a convolutional neural network (CNN) to evaluate the accuracy of machine learning in differentiating optic neuropathies, pseudopapilledema, and normal optic discs, comparing its performance with models such as GoogleNet Inception V3, VGG, and ResNet. Their findings concluded that machine-learning techniques could be combined with fundus photography as an effective method to distinguish PPE from optic disc edema caused by optic neuropathies. The BONSAI research group (Brain and Optic Nerve Study with Artificial Intelligence) trained, validated, and externally tested a deep-learning system (DLS) [17]. They retrospectively collected 15,846 fundus photographs acquired through pharmacologically dilated pupils using various digital cameras, representing diverse ethnic populations. Among these, 14,341 images from 19 sites across 11 countries were used for training and validation, while 1505 images from 5 additional sites were reserved for external testing. The system comprised a segmentation network (U-Net) to localize the optic disc in fundus images and a classification network (DenseNet) to categorize the optic disc as normal, edematous, or other abnormalities. Subsequently, researchers from the same group, Biousse et al. [18], conducted experiments comparing the diagnostic performance of the BONSAI-DLS with neuro-ophthalmologists based solely on optic disc appearance in fundus images. The results demonstrated that the DLS achieved accuracy, sensitivity, and specificity in optic disc classification comparable to or exceeding those of two neuro-ophthalmologists specializing in optic nerve diseases, each with over 30 and 25 years of clinical experience, respectively. Another BONSAI member, Caroline et al. [19], trained a deep-learning system using 2103 dilated fundus photographs from 965 patients to automatically classify papilledema severity. They first trained a U-Net with 6370 fundus images to localize the optic disc region. The trained U-Net was then applied to extract the optic disc area from fundus images, which served as input to a classification network. Finally, a VGGNet-based classifier categorized the optic discs into mild-to-moderate or severe papilledema.

In 2021, Saba et al. [20] proposed a deep-learning system based on U-Net and DenseNet architectures for the automated detection and grading of papilledema. The method involves two primary stages: First, localizing and cropping the optic disc and its surrounding regions in fundus retinal images, which are then fed into a DenseNet for classification as papilledema or normal. Subsequently, Gabor filtering is applied to preprocess images classified as papilledema. These preprocessed images are input into a U-Net to obtain segmented retinal vascular networks. The severity of papilledema is graded as moderate or severe by calculating the vessel discontinuity index (VDI) and vessel discontinuity index to disc proximal (VDIP). Liu Kaiqun et al. [21] from Zhongshan Ophthalmic Center, Sun Yat-sen University, developed and evaluated an artificial intelligence (AI)-based diagnostic system using color fundus images to differentiate optic neuritis (ON) and non-arteritic anterior ischemic optic neuropathy (NAION). They trained and validated the system with annotated color fundus images using the EfficientNet-B0 model. Experimental results demonstrated that the system achieved high sensitivity and specificity in diagnostic performance. Compared to the use of color fundus photography, Ahmed et al. proposed a method based on optical coherence tomography (OCT) images for detecting and grading papilledema. By constructing a cascade architecture that integrates four transfer learning models, they achieved accurate detection and grading of the condition [22].

### 2.2. CNN–Transformer Hybrid Models

Convolutional neural networks (CNNs) and vision Transformers (ViT) [23] represent two dominant deep-learning architectures in computer vision. CNNs excel at extracting local features such as edges and textures through convolutional kernels, making them widely applicable to classification, segmentation, and object detection tasks. However, their inherent limitation in modeling long-range dependencies restricts performance in complex scene understanding tasks. In contrast, ViT employs self-attention mechanisms to capture global features and establish long-range dependencies, demonstrating superior or comparable performance to CNNs across various vision tasks in recent years. Nevertheless, ViT’s requirement for global image interaction leads to significantly increased computational complexity. Additionally, achieving accuracy comparable to CNNs often necessitates ViT models with substantially larger parameter sizes.

To address these challenges, researchers have proposed hybrid architectures that synergistically integrate CNNs and ViT [24]. These frameworks leverage ViT’s self-attention for global context modeling and CNN’s local feature extraction capabilities, thereby enhancing both detail preservation and global semantic understanding to improve vision task performance. Furthermore, reducing input resolution via CNN layers before processing with ViT optimizes computational efficiency while maintaining model accuracy. However, the complexity of hybrid architectures demands a heightened focus on model lightweighting. To meet multi-scenario deployment requirements, researchers have prioritized developing generalizable, easily deployable, and resource-efficient models. Classical examples include lightweight CNN models such as MobileNet [25,26] and ShuffleNet [27], which achieve efficient inference through reduced parameters and computations. With the emergence of hybrid architectures, lightweight CNN-ViT models (e.g., MobileViT [28], EfficientViT [29], Parc-Net [30]) further combine the strengths of both architectures, significantly lowering computational resource consumption without compromising performance, thereby providing efficient solutions for edge-device vision tasks.

### 2.3. Attention Mechanisms in CV

The attention mechanism originates from studies of human visual perception. When humans read books or observe images, they typically focus on key textual content in books or salient regions in images. This mechanism represents a biological strategy for filtering high-value information from massive data through limited processing resources—prioritizing critical elements while suppressing irrelevant content, thereby significantly improving information processing efficiency and accuracy. Deep-learning models incorporating attention mechanisms can enhance the efficiency and precision of image feature extraction, leading to their widespread adoption in computer vision [31]. Based on their operational data domains, attention mechanisms are categorized into four types: channel attention, spatial attention, temporal attention, and hybrid attention.

### 2.4. Wavelet Transforms

Wavelet transform is a powerful and reversible signal processing tool that preserves information integrity without data loss during transformation. Recently, researchers have attempted to integrate wavelet transform into neural network architectures, achieving promising results in computer vision tasks such as image reconstruction and denoising [32,33]. Applying wavelet transform downsampling to input images obtains low-frequency and high-frequency components: the low-frequency components reflect the basic object structures at a coarse-grained level, while the high-frequency components capture the details or local changes within the image at a fine-grained level.

## 3. Methods

### 3.1. Architecture of the WHA-Net

**Figure 2:**
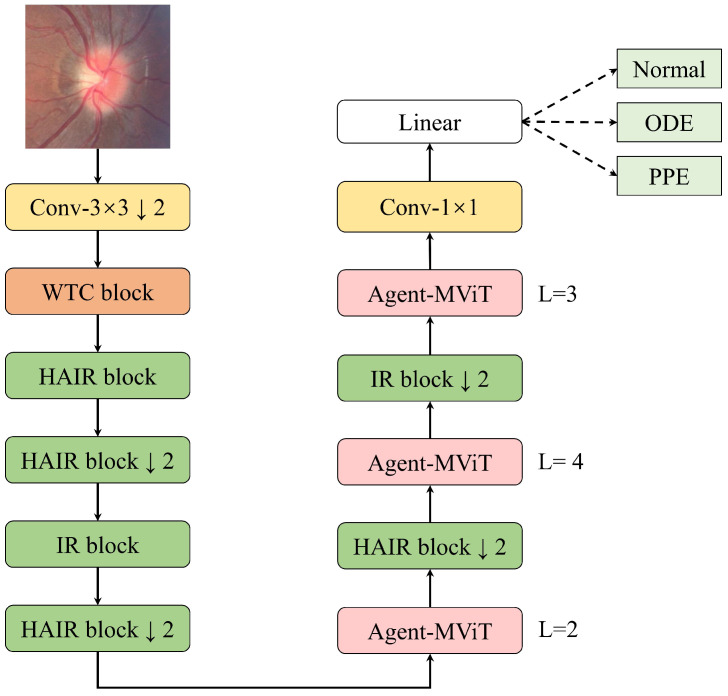
Architecture of the WHA-Net. L represents the number of repeated stacking of the Agent-Transformer module, ↓2 means that the block performs down sampling.

The proposed WHA-Net architecture is designed to enhance classification accuracy for pseudopapilledema while achieving lightweight deployment through reduced parameter counts and computational complexity. As illustrated in Figure 2, WHA-Net first employs a 3 × 3 standard convolutional layer to downsample input RGB fundus images. This is followed by a wavelet convolution (WTC) block to perform convolutional operations and multi-scale feature fusion across different frequency sub-bands, enabling noise reduction while extracting richer feature maps. Subsequently, hybrid attention inverted residual (HAIR) blocks are utilized for feature extraction and local feature construction. The HAIR block integrates hybrid attention mechanisms during dimension expansion, facilitating cross-channel information interaction and localized feature extraction, which effectively models vascular edge characteristics. To balance computational efficiency and feature representation capabilities, partial network layers retain the computationally efficient original inverted residual (IR) structure. The Agent-MViT module, a CNN–Transformer hybrid, concurrently models local and global features in feature maps. Depthwise separable convolution (DWC) handles local feature representation, while the lightweight Agent-Transformer captures long-range dependencies to address the limitations of traditional CNNs in modeling global context. This design enhances the model’s capability to delineate optic disc contours and retinal vascular continuity with minimal computational overhead.

### 3.2. Wavelet Convolution (WTC) Block

In the proposed model, we introduce a wavelet convolution block [34]. Specifically, the input image is first decomposed into four frequency-domain sub-bands via 2D Haar wavelet transform: low-frequency (LL), horizontal high-frequency (LH), vertical high-frequency (HL), and diagonal high-frequency (HH). This is achieved through depthwise convolution using four 2 × 2 filters, as defined in Equation (1), where $f_{LL}$ acts as a low-pass filter while $f_{LH},f_{HL}$, and $f_{HH}$ collectively form high-pass filters. The resulting sub-images maintain 1/4 resolution of the original input.

(1) $$f_{\mathrm{LL}},f_{\mathrm{LH}},f_{\mathrm{HL}},f_{\mathrm{HH}}=\left[\begin{array}{cc} \frac{1}{2} & \frac{1}{2}\\ \frac{1}{2} & \frac{1}{2} \end{array}\right],\left[\begin{array}{cc} \frac{1}{2} & \frac{1}{2}\\ -\frac{1}{2} & -\frac{1}{2} \end{array}\right],\left[\begin{array}{cc} \frac{1}{2} & -\frac{1}{2}\\ \frac{1}{2} & -\frac{1}{2} \end{array}\right],\left[\begin{array}{cc} \frac{1}{2} & -\frac{1}{2}\\ -\frac{1}{2} & \frac{1}{2} \end{array}\right].$$

Lower-resolution sub-images can be obtained through the recursive decomposition of low-frequency components. For each input channel, the convolved feature maps will have four times the number of channels as the input. We employ a two-level wavelet transform, with the transformation operations at each level as follows:

(2) $$\begin{array}{c} \begin{array}{cc} \left[X_{LL},X_{LH},X_{HL},X_{HH}\right]=\mathrm{WT}\left(X\right)=\mathrm{Conv}([f_{LL},f_{LH}, & f_{HL},f_{HH}],X) \end{array} \end{array}$$

(3) $$\begin{array}{c} X_{LL}^{\left(i\right)},X_{LH}^{\left(i\right)},X_{HL}^{\left(i\right)},X_{HH}^{\left(i\right)}=\mathrm{WT}\left(X_{LL}^{\left(i-1\right)}\right) \end{array}$$

where *X* is the input tensor, $X_{LL}^{\left(0\right)}=X$, and *i* is the current level.

**Figure 3:**
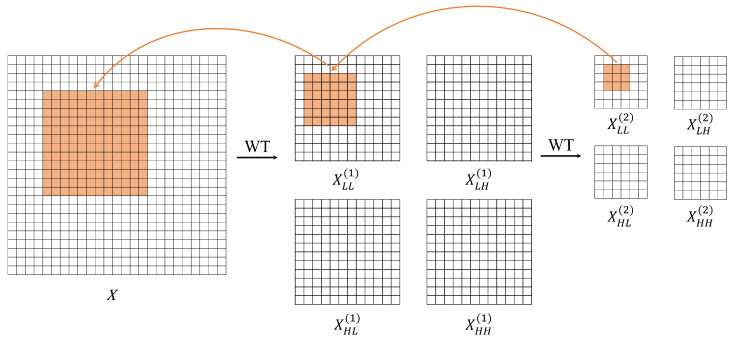
Performing convolution in the second-level wavelet domain results in an effectively larger receptive field.

Following the first-level wavelet transform, 3 × 3 depthwise convolution is performed on the generated frequency sub-bands. Subsequently, second-level wavelet decomposition is applied to the low-frequency components to extract coarser-grained structural representations. With increasing decomposition levels, the low-frequency components progressively exhibit enhanced coarseness, focusing on global structural features (e.g., optic disc boundaries) while demonstrating improved noise robustness. Finally, 3 × 3 depthwise convolution is implemented across all 2-level frequency sub-bands; the operation is shown in Equation (4). By conducting convolutions on low-resolution sub-bands, this approach equivalently achieves large receptive field coverage comparable to bigger convolutional kernels (as Figure 3 illustrated), obtaining information-enriched feature representations without substantial parameter increment.

(4) $$Y_{LL}^{\left(i\right)},Y_{LH}^{\left(i\right)},Y_{HL}^{\left(i\right)},Y_{HH}^{\left(i\right)}=\mathrm{Conv}\left(W^{\left(i\right)},\left(X_{LL}^{\left(i\right)},X_{LH}^{\left(i\right)},X_{HL}^{\left(i\right)},X_{HH}^{\left(i\right)}\right)\right)$$

The input feature maps are recursively decomposed and subjected to convolution operations, after which the feature maps need to be reconstructed to their original pre-decomposition scale. This module employs transposed convolution as the inverse wavelet transform method for upsampling. In this module, the second-level sub-bands undergo inverse transformation to match the dimensions of the first-level sub-bands. To aggregate feature maps from different frequency domains, the inversely transformed sub-bands are element-wise summed with the convolution-processed first-level low-frequency sub-band, while other first-level sub-bands remain unchanged. Finally, the processed first-level feature maps undergo inverse wavelet transformation, restoring the feature map resolution to the input dimensions. The operational procedure is formalized in Equation (6).

**Figure 4:**
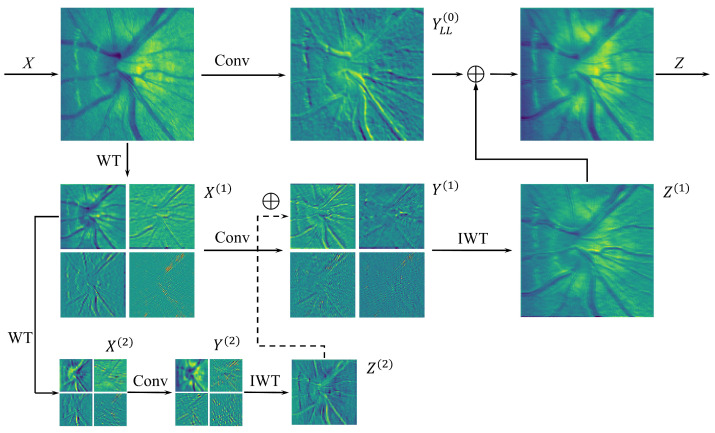
An example of the wavelet convolution (WTC) operation on a single channel.

The WTC module architecture comprises two processing branches: one branch extracts original scale features $Y_{LL}^{\left(0\right)}$ through 1 × 1 convolution operations on input *X*, as described in Equation (5), while the other branch ultimately aggregates multi-frequency domain features $Z^{\left(1\right)}$ through wavelet transformation and convolution operations. These two feature components are subsequently fused to constitute the final output *Z* of the WTC module, as mathematically expressed in Equation (7). The comprehensive workflow of WTC operations is illustrated in Figure 4.

(5) $$\begin{array}{c} Y_{LL}^{\left(0\right)}=\mathrm{Conv}\left(W^{\left(0\right)},X_{LL}^{\left(0\right)}\right), \end{array}$$

(6) $$\begin{array}{c} Z^{\left(i\right)}=\mathrm{IWT}\left(Y_{LL}^{\left(i\right)}+Z^{\left(i+1\right)},Y_{LH}^{\left(i\right)},Y_{HL}^{\left(i\right)},Y_{HH}^{\left(i\right)}\right) \end{array}$$

(7) $$Z=Y_{LL}^{\left(0\right)}+Z^{\left(1\right)}$$

where *W* is the weight tensor of a 3×3 depth-wise kernel. For *l*-level wavelet transform, $Z^{\left(l+1\right)}=0$. The ’+’ symbol denotes the element-wise summation of feature maps.

Compared to the single-scale filtering of standard convolution, the WTC module explicitly decouples distinct frequency-domain features, enhancing the model’s differentiated representation capability for structural contours (low-frequency) versus edge/texture details (high-frequency), which in this task directly corresponds to optic disc morphology and vessel characteristics in retinal imaging.

### 3.3. Hybrid Attention Inverted Residual (HAIR) Block

**Figure 5:**
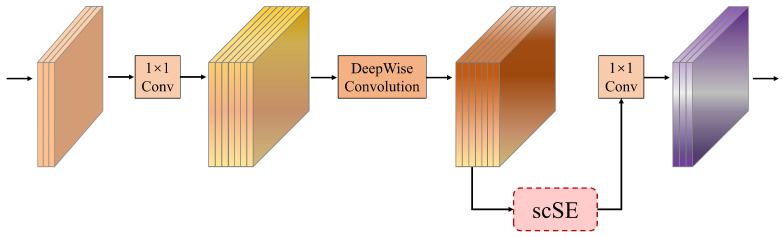
Hybrid attention inverted residual (HAIR) block structure.

ODE is radiologically characterized by peri-papillary exudative changes, vascular border blurring, and hemorrhagic lesions. To enhance the model’s capability in extracting discriminative features for PPE identification, we integrate an improved spatial-channel hybrid attention mechanism (scSE) [35] into the classical inverted residual (IR) architecture, constructing a hybrid attention inverted residual (HAIR) block. As illustrated in Figure 5, this improved design introduces a lightweight attention mechanism, achieving significant enhancement in local vascular edge and pathological exudative feature extraction with only minimal computational overhead.

**Figure 6:**
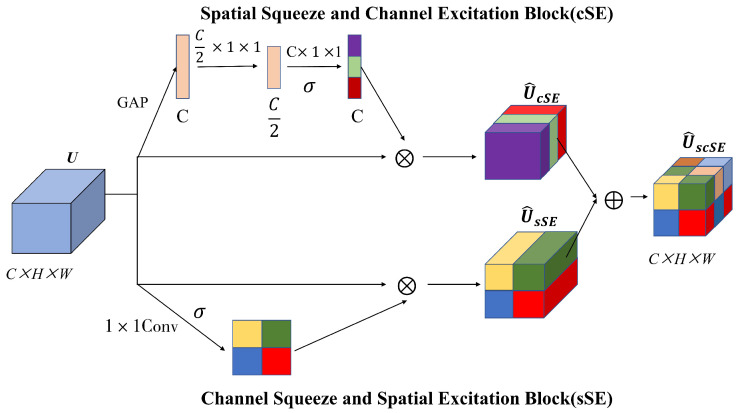
The structure of scSE module. σ represents Hard-Sigmoid activation function.

The scSE module is composed of the spatial squeeze and channel excitation (cSE) block and the channel squeeze and spatial excitation (sSE) block connected in parallel, as illustrated in Figure 6. The cSE module compresses the spatial dimensions of the feature map through global average pooling, followed by two 1 × 1 convolutional layers (with a SiLU activation function interposed between them) to learn inter-channel dependencies. Channel-wise weights are generated via Hard-Sigmoid normalization and multiplied element-wise with the original features along the channel dimension, thereby enhancing the responses of critical channels. The sSE module reduces the number of input feature channels to 1 through a 1 × 1 convolution, then generates a spatial attention map via Hard-Sigmoid activation across spatial dimensions. This map is element-wise multiplied with the original features at corresponding spatial positions to emphasize crucial regions. The final output integrates features enhanced by both channel and spatial attention mechanisms, strengthening task-relevant feature representation. This dual-attention fusion strategy demonstrates superior adaptability to complex scenarios compared to single-attention mechanisms.

The HAIR block implementation follows three stages: First, channel expansion via 1 × 1 convolution increases input feature channels to target dimensions. Next, depthwise separable convolution extracts spatial features, with scSE hybrid attention modules embedded after each depthwise layer to enable simultaneous focus on critical channels and key spatial regions. Finally, channel reduction through 1 × 1 convolution is combined with non-linear activation functions to minimize information loss. Specifically, shallow layers employ SiLU activation, while high-channel-dimension layers utilize Hard-Swish activation. Compared to ReLU and Swish, Hard-Swish (achieving Swish-like characteristics through piecewise linear approximation) maintains non-linear representation capacity while significantly reducing computational complexity.

### 3.4. Agent-MViT Block

The proposed Agent-MViT block in this section draws inspiration from the MobileViTv3 architecture [36], employing a hybrid design that combines local feature representation units with global feature representation units. This integration leverages the spatial inductive bias of CNNs and the global dependency modeling capability of ViT, while effectively reducing computational complexity through an agent-based attention mechanism.

**Figure 7:**
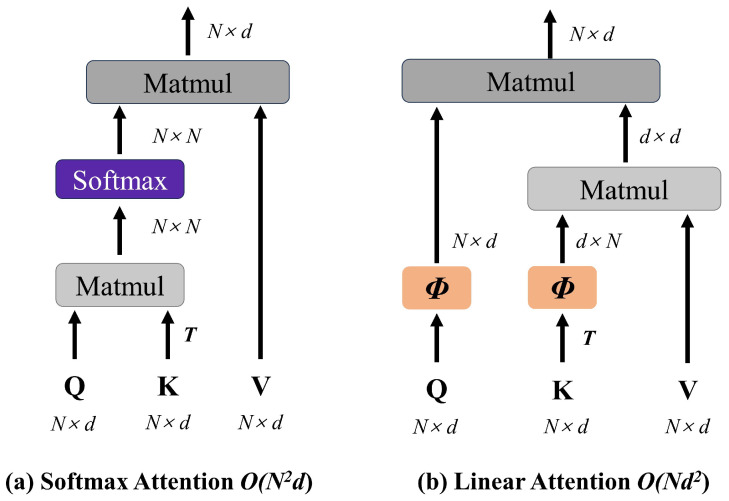
Traditional self attention. (**a**) Softmax attention, (**b**) Linear attention.

The Transformer in MobileViT employs Softmax-scaled dot-product self-attention to capture contextual relationships among N tokens. The Softmax attention mechanism first applies linear transformations to the input *X* using weight matrices to generate Query (*Q*), Key (*K*), and Value (*V*) matrices, as formalized in Equation (8). Subsequently, similarity scores between the *Q* and *K* matrices are computed. To mitigate gradient vanishing caused by excessively large dot-product values, these scores are scaled by a factor determined by the single-head vector dimension $d_{k}$. The scaled similarity score matrix is then row-wise normalized using the Softmax function to produce the attention weight matrix, which represents the probability of each position attending to others. Finally, the output is generated by a weighted summation of the *V* matrix using these attention weights, as detailed in Equation (9), with the overall workflow illustrated in Figure 7a.

(8) $$Q=XW_{Q},K=XW_{K},V=XW_{V}$$

(9) $$\mathrm{Attention}\left(Q,K,V\right)=\mathrm{Softmax}\left(\frac{QK^{⊤}}{\sqrt{d_{k}}}\right)V$$

However, Softmax attention incurs increased computational and temporal costs due to $O(N^{2})$ time complexity. For ViT models with large token sequences, this quadratic complexity becomes a significant drawback as token length grows. Furthermore, the multi-head attention mechanism—which processes batched matrix multiplications and Softmax computations—imposes substantial computational and memory burdens, hindering deployment on devices with limited computational and memory resources. To address this, researchers have proposed linear attention mechanisms that apply direct linear projections to *Q* and *K* while eliminating the Softmax function, thereby reducing computational complexity [37], as depicted in Figure 7b. Nevertheless, such coarse-grained approximations result in substantial degradation of model representational capacity.

**Figure 8:**
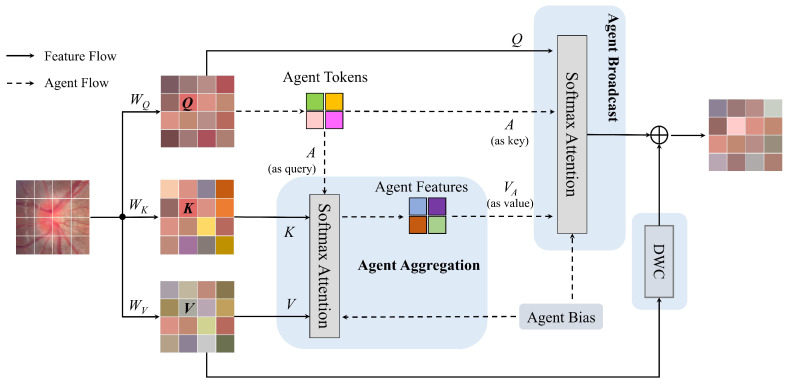
An illustration of agent attention module.

To address the aforementioned limitations, we integrate the agent attention module proposed by Han et al. in 2024 [38]. This module comprises three core components: a pure agent attention unit, agent bias terms, and a depthwise convolutional (DWC) block, as shown in Figure 8, synergistically combining the high representational capacity of Softmax attention with the low computational complexity of linear attention.

**Figure 9:**
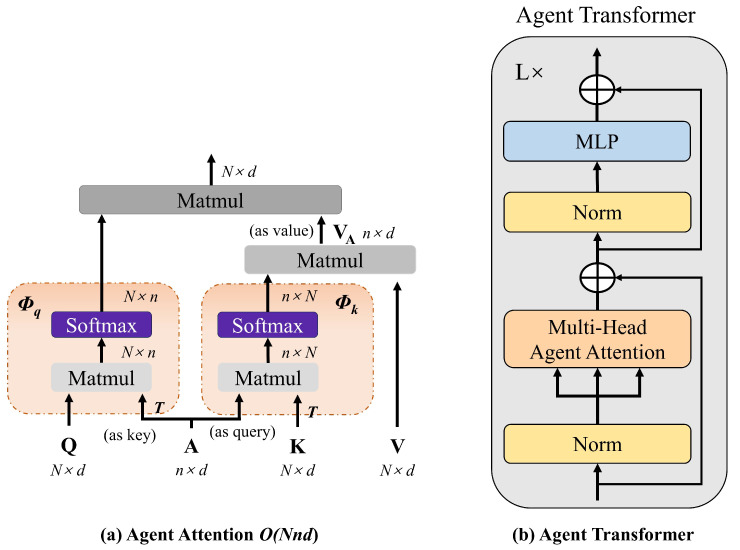
Agent attention and agent Transformer.

The agent attention mechanism implements two Softmax attention operations: First, agent tokens (*A*) serve as query vectors to perform initial Softmax attention with keys (*K*) and values (*V*) (agent feature aggregation phase), generating agent features $V_{A}$. Subsequently, using agent tokens *A* as keys and $V_{A}$ as values, a second Softmax attention operation (agent feature broadcasting phase) maps global agent features to each query token through the query matrix *Q*, as shown in Figure 9a.

When agent tokens are generated solely via feature pooling, their spatial positional modeling capability becomes constrained, leading to attention focus misalignment. To mitigate this, learnable positional bias terms are incorporated to enhance the localization accuracy of agent tokens during aggregation and broadcasting, thereby improving overall model performance. Furthermore, to counter potential feature diversity reduction in agent attention, depthwise convolution (DWC) is applied to the value matrix *V* for spatial feature enhancement, with residual connections fusing these features into the attention output to preserve model representational capacity. The calculation flow of the agent attention module is shown in Equation (10).

(10) $$\begin{array}{cc} Z= & \;\sigma \left(QA^{T}\;\;+\;B_{2}\right)\;\sigma \left(AK^{T}\;\;+\;B_{1}\right)\;V+\mathrm{DWC}\left(V\right), \end{array}$$

where $Q,K,V\in R^{N\times d}$, $A\;\in \;R^{n\times d}$ denote query, key, value, and agent matrices; $B_{1}\;\in \;R^{n\times N}$ and $B_{2}\;\in \;R^{N\times n}$ are agent biases; and σ(·) represents Softmax function.

**Figure 10:**
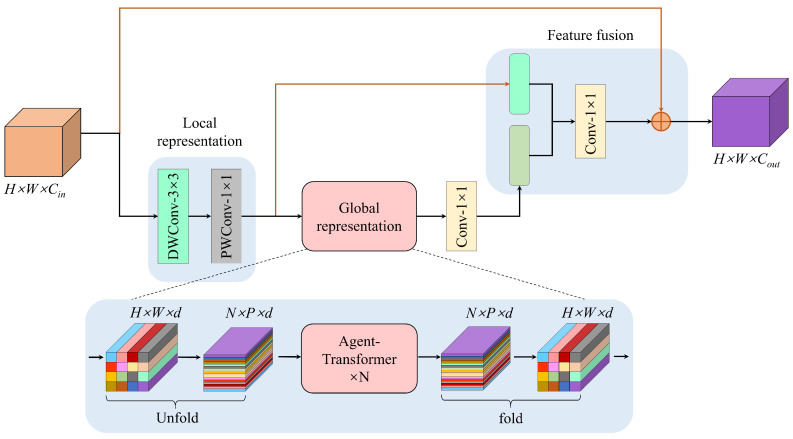
Architecture of the Agent-MViT block.

The Agent-MViT module processing pipeline consists of three key stages: Firstly, local feature modeling is performed through a depthwise separable convolutional (DWC) layer, followed by 1 × 1 convolution to adjust channel dimensions to meet Agent-Transformer’s input requirements. The Agent-Transformer module primarily comprises a multi-head agent attention mechanism and a multi-layer perceptron (MLP), which are sequentially connected via residual connections and layer normalization (Norm), as depicted in Figure 9b. During global representation learning, an unfold operation reshapes the feature map into the standard input format for agent attention. After global relationship modeling via Agent-Transformer, a fold operation restores the original spatial dimensions. Finally, 1 × 1 convolution reverts channel dimensions to match the initial input. The complete workflow is detailed in Figure 10.

## 4. Experiments and Results

### 4.1. Dataset

The training, validation, and test data in this study consisted of color fundus images captured by fundus cameras. To improve model generalizability, we collected and utilized fundus images from multiple sources, including the collection by neuro-ophthalmologist Dr. William F. Hoyt (United States) [39], Kim’s Eye Hospital (Republic of Korea) [40], and publicly available datasets such as RFMiD (India) [41], ODIR (China) [42], and G1020 (Germany) [43]. To ensure age diversity, data from the Ophthalmology Department of Children’s Hospital of Fudan University (China) were also included. However, detailed demographic information (e.g., age, gender, ethnicity) was not uniformly available across all datasets due to limitations in original data collection protocols. All images were clinically annotated by ophthalmologists based on optic disc morphology, visual function assessments, medical history, and auxiliary examinations. If the labeling of a certain image in the public datasets did not clearly specify whether it is ODE or PPE, we did not include it. In addition, two ophthalmologists with years of clinical experience were involved in the screening process. Fundus images that did not fully include the optic disc region were also excluded. After excluding low-quality or diagnostically ambiguous images, 1793 images were retained, comprising 895 normal optic discs, 485 optic disc edema (ODE), and 413 pseudopapilledema (PPE). The dataset was split into 90% for training and 10% for testing, with the training data further split into training and validation sets at a 3:1 ratio for model parameter optimization and hyperparameter tuning, respectively. The sample number distribution of the dataset is shown in Table 1.

**Figure 11:**
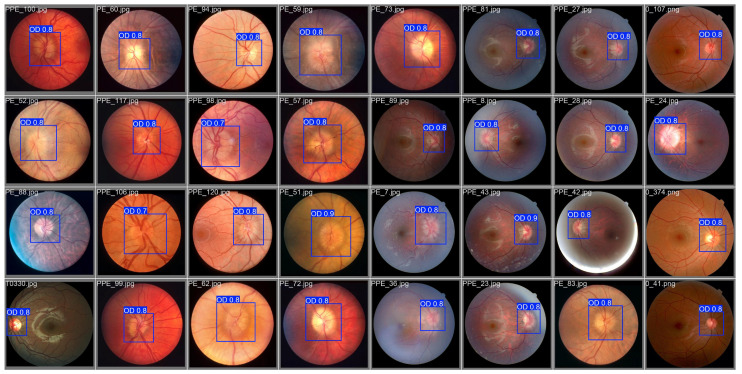
The performance of the trained YOLOv8Nano model on the optic disc detection task across a multi-source dataset.

**Figure 12:**
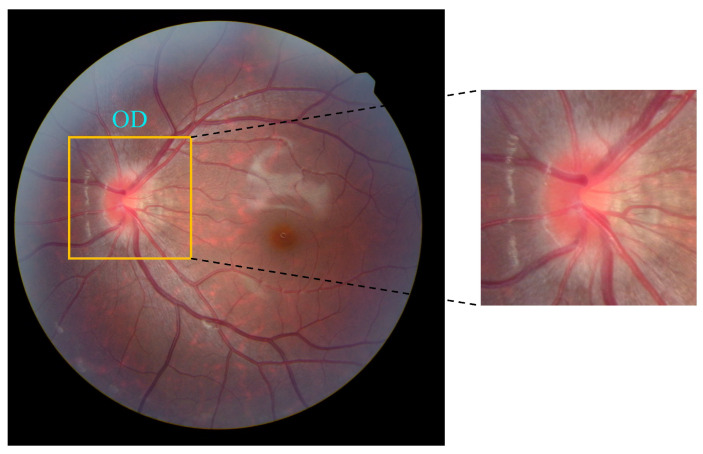
Detecting and cropping optic disc on fundus image.

Since the key features for identifying papilledema in images are concentrated in the optic disc and its adjacent regions—including optic cup status, disc contour, and continuity of main vessels—while original fundus images may contain task-irrelevant features like macular areas and peripheral retinal vessels, we employed a pretrained YOLOv8Nano model to detect and crop the optic disc region as input for the classification model. We constructed and annotated a multi-source optic disc detection dataset containing 400 images. On the validation set, the model achieved a precision of 99.4%, a recall of 98.3%, and an mAP50 of 99%, demonstrating its effectiveness in optic disc detection, as shown in Figure 11. This approach reduces feature redundancy, allowing the model to focus on learning disc-related characteristics, improves feature extraction efficiency, and mitigates potential classification errors caused by quality issues (e.g., blurring, uneven illumination) in other fundus regions, as shown in Figure 12.

Additionally, given our dataset’s limited size, we applied data augmentation to the training set to prevent underfitting and enhance model robustness across varying image qualities. Augmentation methods included random rotation, brightness/contrast adjustment, and Gaussian noise addition, expanding the training set from 1209 to 4836 images. During training, we further implemented random cropping and horizontal flipping to simulate real-world variations, thereby improving inference accuracy.

### 4.2. Evaluation Metrics

Evaluation metrics serve as essential tools for assessing classification model performance, with different indicators reflecting specific aspects of model capabilities. This study employs four metrics to evaluate network performance: Accuracy, Precision, Recall, and Specificity. Accuracy is defined as the proportion of correctly classified samples relative to the total sample size, reflecting the model’s overall predictive capability. Precision represents the ratio of true positive samples among those predicted as positive, measuring the reliability of positive predictions. Recall indicates the proportion of actual positive samples correctly identified, demonstrating the model’s detection capacity for positive cases. Specificity denotes the ratio of true negative samples correctly classified, characterizing the model’s discriminative power for negative instances. All metrics are calculated based on statistical quantities of true positives (TP), true negatives (TN), false positives (FP), and false negatives (FN).

(11) Accuracy=(TP+TN)/(TP+TN+FN+FP)

(12) Precision=TP/(TP+FP)

(13) Recall=TP/(TP+FN)

(14) Specificity=TN/(TN+FP)

### 4.3. Experimental Settings

In implementation, we utilized the PyTorch 2.2.0 framework to construct the proposed model and conduct experiments. All experiments were performed on a Windows 11 workstation equipped with an Intel Core i9-13900 CPU, 64 GB RAM, and a 24GB NVIDIA GeForce RTX 4090 GPU. The entire framework was trained in an end-to-end manner. Input images were resized to 256 × 256 pixels. The AdamW optimizer was employed for parameter optimization, with a weight decay coefficient of 0.02, batch size of 64, and initial learning rate of 0.0005. A cosine annealing schedule with a learning rate scaling factor (lrf) of 0.2 was adopted for the automatic learning rate adjustment. The cross-entropy function was selected as the classification loss function.

### 4.4. Classification Performance of WHA-Net

We comprehensively evaluated the model’s predictive performance on three-class classification tasks (normal optic discs, ODE, and PPE) using the test set. As shown in Table 2, WHA-Net demonstrated outstanding performance across all categories and overall metrics: The model achieved 100% accuracy in classifying normal optic discs, confirming its robust recognition capability for physiological disc features. Both ODE and PPE attained an accuracy of 0.9669, with PPE showing a recall of 0.9524, indicating superior detection performance for PPE cases. However, the relatively lower ODE recall rate was primarily attributed to morphological similarities between early-stage ODE and PPE cases, leading to misclassification of some samples as PPE.

### 4.5. Comparison with Other Methods

To validate the performance of our proposed model, we conducted comparative experiments with recently developed lightweight CNN models (DenseNet-169 [44], EfficientNetV2 [45], ShuffleNetV2, MobileNetV4 [46], and RepViT [47]) that have achieved state-of-the-art results in image classification tasks, along with CNN–Transformer hybrid architectures (MobileViTv3, EfficientViT). For fairness, all models were trained on identical datasets, with hyperparameters optimized to achieve peak validation accuracy. The resulting configurations were then evaluated on the same test set. Metrics including Accuracy, Precision, Recall, and Specificity are reported as average values across all three classes. Additionally, parameter counts and FLOPs were included as supplementary efficiency indicators.

**Figure 13:**
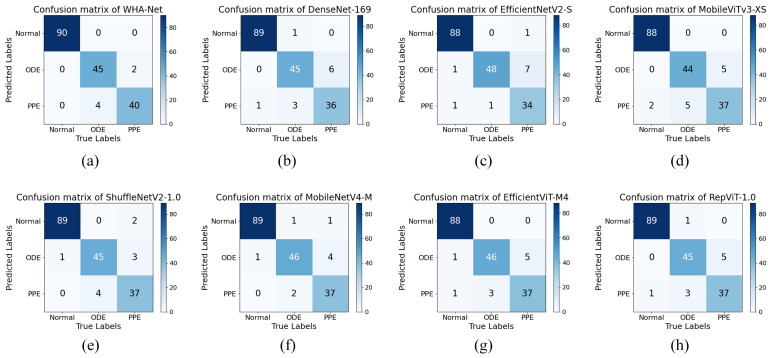
Confusion matrix on test set. Detailed description as shown at the top of each subfigure.

As detailed in the comparative experiments shown in Table 3, WHA-Net achieved robust performance, with an average accuracy of 97.79%, mean precision of 95.55%, mean recall of 95.69%, and mean specificity of 98.53%. The experimental results demonstrate that, compared to existing methods, our model attained optimal classification performance across all evaluation metrics. The mean accuracy surpassed the second-ranked MobileNetV4-M by 1.11%. DenseNet-169 and EfficientNetV2-S, which adopt conventional convolutional stacking architectures, exhibited significantly higher parameter counts and computational costs (FLOPs) than other comparative models but showed suboptimal classification performance on this dataset. Notably, while ShuffleNetV2 demonstrated strong potential for edge device deployment due to its minimal parameter size and computational requirements, its limited model capacity and inadequate long-range dependency modeling capability constrained feature representation effectiveness, leading to reduced classification accuracy. This is clearly observed in the confusion matrix, where ShuffleNetV2 frequently misclassified pseudopapilledema (PPE) cases as normal optic discs, as shown in Figure 13e.

WHA-Net achieves an exceptional balance between low complexity and high accuracy, highlighting its significant advantages in pseudopapilledema classification tasks. Through its carefully designed hybrid architecture, WHA-Net maintains relatively low parameter counts and computational costs while achieving state-of-the-art classification accuracy, fully validating its efficiency and practicality in resource-constrained scenarios. This balance not only enables WHA-Net to better adapt to computational limitations in real-world medical applications but also ensures high reliability and stability in classification tasks.

### 4.6. Explaining Model with Grad-CAM

To more directly demonstrate the superiority of WHA-Net, we employed Grad-CAM (gradient-weighted class activation mapping) to visualize how the model makes classification decisions. Grad-CAM generates heatmaps by backpropagating the gradient information of target classes and fusing it with feature maps through weighted integration. In these heatmaps, highlighted regions indicate key feature locations the model focuses on during prediction, with color gradients from blue to red representing increasing attention intensity.

**Figure 14:**
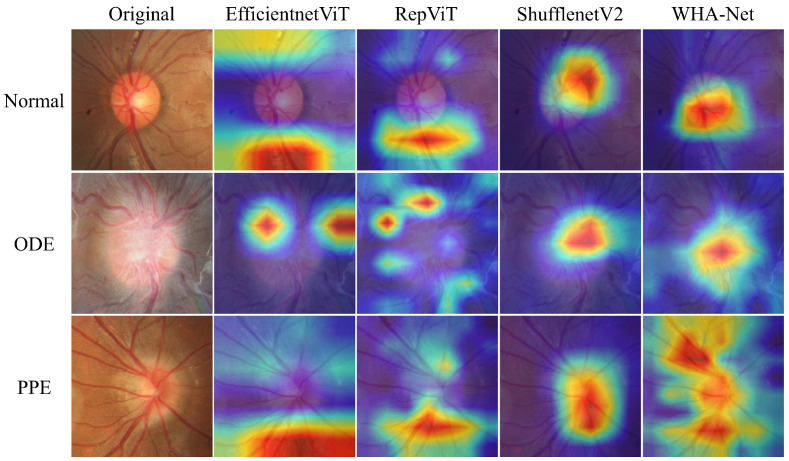
Grad-CAM visualization of different methods.

Three representative test cases were selected, and comparative analyses were conducted with three high-performing models: EfficientViT, RepViT, and ShuffleNetV2. Gradients were calculated based on our specified target classes, allowing clear visualization of the regions each model prioritized during predictions. As shown in Figure 14, EfficientViT and RepViT exhibited dispersed attention patterns across the images compared to other models. While ShuffleNetV2 primarily focused on the optic disc region, it demonstrated limited discriminative capability for pseudopapilledema. In contrast, when classifying pseudopapilledema, WHA-Net’s attention mechanism not only accurately concentrated on the optic disc area but also effectively captured the morphological continuity of surrounding vasculature, enhancing feature discriminability. Across all three cases, WHA-Net consistently demonstrated superior local-global attention coordination capability.

### 4.7. Ablation Study

WHA-Net was designed based on architectural inspiration from MobileViTv3. We first replaced the standard Transformer module in MobileViTv3 with an agent attention module while retaining other configurations, naming the modified architecture Agent-MViTv3. Under identical hyperparameters and training protocols, comparative experiments between the original MobileViTv3 and Agent-MViTv3 were conducted, with evaluation metrics including validation accuracy, parameter count, and computational complexity (FLOPs). As shown in Table 4, the modified model reduced FLOPs from 1.0 G to 889 M (11.10% decrease), with only a 5.95% increase in parameters, confirming the effectiveness of agent attention in lowering computational complexity. Notably, the modified model exhibited a slight decline in validation accuracy, necessitating subsequent architectural refinements to enhance performance.

As described in Section 3, the proposed network architecture integrates three core components: the WTC block, HAIR block, and Agent-MViT block. To validate the effectiveness of these modules in improving classification performance and reducing computational complexity, we conducted phased ablation studies: starting with a baseline model (Agent) derived from Agent-MViTv3 with partial structural removal, followed by sequential integration of the WTC block, HAIR block, and their combination. All comparative models shared identical training parameters and environmental configurations.

Table 5 demonstrates that introducing the WTC block alone maintained comparable parameter counts to the baseline while increasing computational costs by 6.12 M FLOPs, resulting in a 0.5% validation accuracy improvement (96.77%→97.27%). Isolated HAIR block integration increased parameters by 0.206 M and FLOPs by 5.76 M, achieving a 0.26% accuracy gain (96.77%→97.02%). The synergistic combination of both modules delivered a 0.75% accuracy boost to 97.52%, significantly outperforming individual module implementations and confirming the efficacy of the modular co-design strategy.

## 5. Discussion

**Figure 15:**
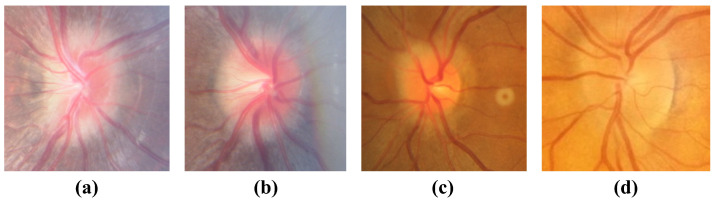
Images of ODE misclassified as PPE. (**a**–**c**) Early stages of ODE, (**d**) Image blur.

**Figure 16:**
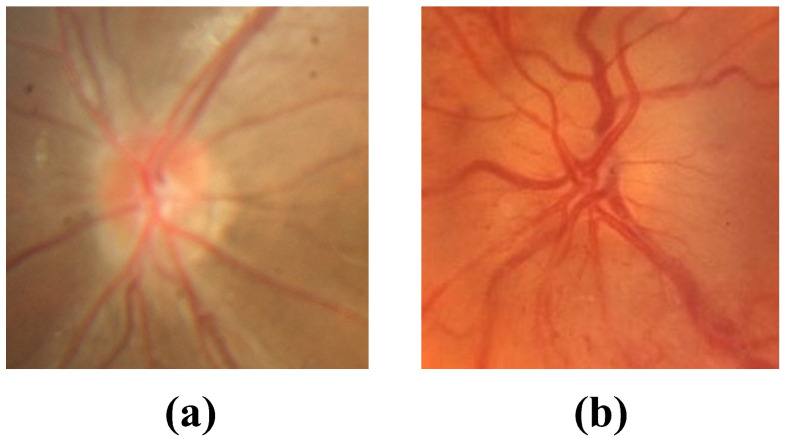
Images of PPE misclassified as ODE. (**a**) Image blur, (**b**) Buried drusen.

While the model effectively distinguishes optic disc edema (ODE) from pseudopapilledema (PPE) in most cases, classification challenges persist in morphologically ambiguous cases. Fundus images from such cases exhibit high similarity in color, texture, and anatomical structure. In the early stages of optic disc edema, fundus images show clear blood vessels, with a small range of optic disc swelling and a low degree of elevation, which may be misclassified as PPE, as shown in Figure 15a–c. In images with low clarity, the model cannot effectively recognize the corresponding features, leading to misclassification, as shown in Figure 15d and Figure 16a. However, the classification confidence scores for these images are relatively low, with Figure 15d classified as PPE with a confidence of 0.7954 and Figure 16a classified as ODE with a confidence of 0.5062, both below 95%. A threshold can be set to alert physicians for additional diagnostic evaluation. In addition, buried optic disc drusen can also cause optic disc margin blurring and elevation, resembling the appearance of disc swelling, as shown in Figure 16b, leading the model to misclassify it as ODE. To address such cases, OCT or OCTA images will be incorporated into the later stages for accurate diagnosis.

Furthermore, the scarcity of these borderline cases in the training dataset limits the model’s ability to learn nuanced distinctions. Enhancing classification accuracy and reliability for these edge cases is critical for clinical translation. We propose the following optimization strategies:

1. Expand the challenging case database: Systematically collect fundus images covering all ODE progression stages, with emphasis on early-stage cases exhibiting overlapping features with PPE to enhance the model’s sensitivity to subtle discriminative patterns.
2. Multimodal data integration: Incorporate multimodal data such as optical coherence tomography (OCT) images [48] and clinical history text [49] for joint training and prediction, leveraging cross-modal feature complementarity to improve diagnostic accuracy (will increase the system complexity).
3. Confidence threshold alert: When prediction probability falls below 95%, activate a “Low Diagnostic Confidence - Clinical Review Recommended” prompt, guiding clinicians and patients toward supplementary diagnostic verification.

## 6. Conclusions

This study proposes a novel efficient hybrid model, WHA-Net, for the automated diagnosis of pseudopapilledema. The core architecture integrates three key components: wavelet convolution (WTC) block, hybrid attention inverted residual (HAIR) block, and agent attention MViT (Agent-MViT) block. The effectiveness of each module is systematically validated through ablation studies, with comprehensive comparisons against mainstream state-of-the-art models. Experimental results demonstrate that WHA-Net achieves an optimal balance between computational complexity and classification accuracy, providing an efficient and reliable solution for pseudopapilledema diagnosis with substantial clinical application potential.

## Figures and Tables

**Table 1:** Sample number distribution of dataset.

Label	Normal	ODE	PPE
Training set	616	330	263
Validation set	189	106	108
Test set	90	49	42
Total	895	485	413

**Table 2:** Classification performance of WHA-Net.

Label	Acccuracy	Precision	Recall	Specificity
Normal	1.0	1.0	1.0	1.0
ODE	0.9669	0.9574	0.9184	0.9848
PPE	0.9669	0.9091	0.9524	0.9712
Average	0.9779	0.9555	0.9569	0.9853

**Table 3:** Performance comparison with state-of-the-art models.

Model	Acccuracy	Precision	Recall	Specificity	Params (M)	Flops
DenseNet-169	0.9595	0.9238	0.9215	0.9716	12.49	3.44 G
EfficientNetV2-S	0.9595	0.9301	0.9223	0.9713	20.18	6.74 G
ShuffleNetV2 (x1.0)	0.9632	0.9329	0.9294	0.9730	**1.26**	**197 M**
MobileViTv3-xs	0.9558	0.9130	0.9189	0.9706	1.85	1.0 G
EfficientViT-M4	0.9632	0.9290	0.9325	0.9752	8.42	300 M
MobileNetV4-M	0.9668	0.9429	0.9362	0.9752	8.44	1.11 G
RepViT (m1.0)	0.9632	0.9304	0.9294	0.9741	6.41	1.13 G
**WHA-Net (Ours)**	**0.9779**	**0.9555**	**0.9569**	**0.9853**	2.15	810 M

**Table 4:** Ablation study for agent attention.

Model	Valid_Acc (%)	Parameters (M)	Flops
MobileViTv3	97.27	1.85	1.0 G
Agent- MViTv3	97.02	1.96 (+5.95%)	889 M (−11.10%)

**Table 5:** Ablation study results of WHA-Net modules.

Model	Valid_Acc (%)	Parameters (M)	Flops (M)
Agent (baseline)	96.77	1.939	797.93
Agent + WTC	97.27	1.941	804.05
Agent + HAIR	97.02	2.145	803.69
WHA-Net (ours)	97.52	2.147	809.81

## Data Availability

The data that support the findings of this study are available from the author upon reasonable request.
